# Supplementary material for: Renal graft function in transplanted patients correlates with CD45RC T cell phenotypic signature
Source: PLoS One. 2024 Mar 21;19(3):e0300032. doi: 10.1371/journal.pone.0300032 (PMC10956768; doi:10.1371/journal.pone.0300032)
Supplement: S1 Fig — (A) Violin plots showing the age of STA and REJ recipients of kidney transplantation included in the cohort analyzed before or after transplantation. Solid line: median, dotted lines: quartiles. (B) Incidence of transplant rejection in the younger half (black line) or older half (blue line) of patients. Median = 52 year-old. (C) Incidence of graft rejection in recipients grafted with sex-matched or mismatched donor. F = female, M = male. (D) Incidence of graft rejection in patients that display more (blue line) or less (black line) than 54.30% or 39.10% CD4+ T cells in PBMCs before (left) or after (right) transplantation respectively. (E) Incidence of graft rejection in patients that display more (blue line) or less (black line) than 20.70% or 26.20% CD8+ T cells in PBMCs before (left) or after (right) transplantation respectively. (C-E) n = 45; Log Rank (Mantel Cox) test, ns. (F) Correlation analysis of the frequency of CD4+ (left) and CD8+ (right) T cells with time post-transplantation. n = 93 samples. (G) Correlation analysis of the frequency of CD45RChi cells in CD4+ (left) and CD8+ (right) T cells after transplantation with time post-transplantation free of acute rejection (AR) episodes. n = 46. (F-G) Thick line = linear regression, thin lines = 95% confidence. (H) Incidence of graft rejection in patients that display more (blue line) or less (black line) than 7.7% CD45RClo/-FOXP3+ cells in T cells before transplantation. Log Rank (Mantel Cox) test, ns. (PDF) [file pone.0300032.s001.pdf]

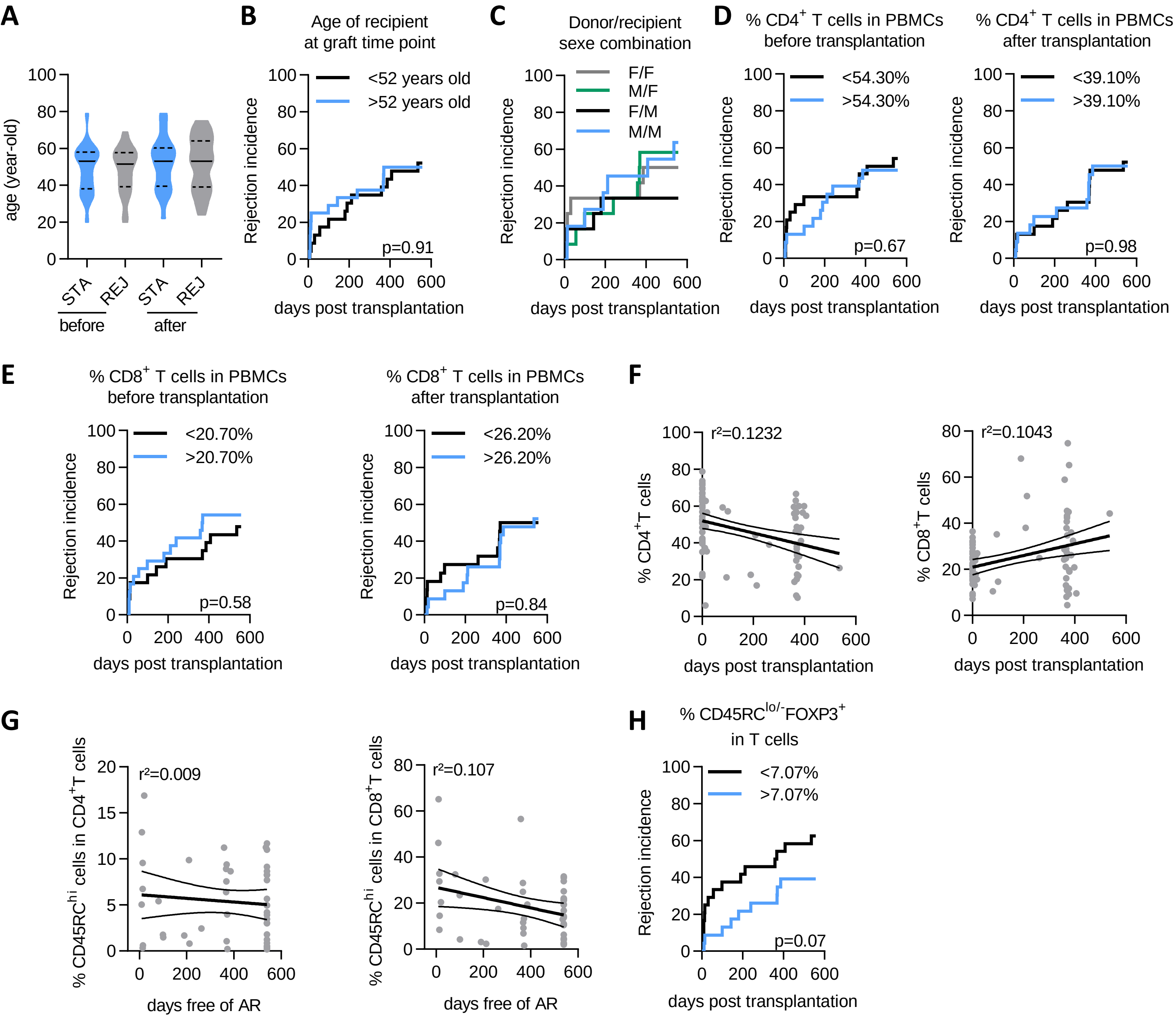

S1 Fig. Characteristics of the transplanted patient cohort.

(A) Violin plots showing the age of STA and REJ recipients of kidney transplantation included in the cohort analyzed before or after transplantation. Solid line: median, dotted lines: quartiles. **(B)** Incidence of transplant rejection in the younger half (black line) or older half (blue line) of patients. Median= 52 year-old. **(C)** Incidence of graft rejection in recipients grafted with sex-matched or mismatched donor. F=female, M=male. **(D)** Incidence of graft rejection in patients that display more (blue line) or less (black line) than 54.30% or 39.10% CD4⁺ T cells in PBMCs before (left) or after (right) transplantation respectively. **(E)** Incidence of graft rejection in patients that display more (blue line) or less (black line) than 20.70% or 26.20% CD8⁺ T cells in PBMCs before (left) or after (right) transplantation respectively. **(C-E)** n=45; Log Rank (Mantel Cox) test, ns. **(F)** Correlation analysis of the frequency of CD4⁺ (left) and CD8⁺ (right) T cells with time post-transplantation. n=93 samples. **(G)** Correlation analysis of the frequency of CD45RC^{hi} cells in CD4⁺ (left) and CD8⁺ (right) T cells after transplantation with time post-transplantation free of acute rejection (AR) episodes. n=46. **(F-G)** Thick line = linear regression, thin lines = 95% confidence. **(H)** Incidence of graft rejection in patients that display more (blue line) or less (black line) than 7.7% CD45RC$^{lo/-}$FOXP3⁺ cells in T cells before transplantation. Log Rank (Mantel Cox) test, ns.
