# Supplementary material for: Renal graft function in transplanted patients correlates with CD45RC T cell phenotypic signature
Source: PLoS One. 2024 Mar 21;19(3):e0300032. doi: 10.1371/journal.pone.0300032 (PMC10956768; doi:10.1371/journal.pone.0300032)
Supplement: S2 Fig — (A-B) Frequency of CD28- cells in CD8+CD45RClo/- FOXP3- (A) and FOXP3+ (B) T cells in STA (n = 11) and REJ (n = 13) patients before and after transplantation. Wilcoxon matched-pairs signed rank test for time comparison and Mann Whitney test for groups comparison, *p<0.05. (C) Frequency of CD28- cells in CD8+CD45RChi T cells. STA, n = 22; REJ, n = 24. (D) Incidence of allograft rejection for patients having more (blue line) or less (black line) than 0.3% CD28- cells in CD8+CD45RChi T cells. (E) Frequency of HLA-DR+ cells in FOXP3- and FOXP3+ CD4+CD45RClo/- T cells. STA, n = 20; REJ, n = 23. Mann Whitney test, **p<0.01. (F-G) Incidence of allograft rejection for patients having more (black line) or less (blue line) than 4.98% HLA-DR+ cells in CD4+CD45RClo/- FOXP3- T cells (F) or 20% HLA-DR+ cells in CD4+CD45RClo/- FOXP3+ T cells (G). (H) Incidence of allograft rejection for patients treated with Simulect (n = 32) or ATG (n = 14) as induction. (I-L) Incidence of allograft rejection for patients treated with Simulect and having more (blue line) or less (black line) than 52.21% CD28- cells in CD8+CD45RClo/-FOXP3- T cells (I, n = 17 STA and 15 REJ), 50.85% CD28- cells in CD8+CD45RClo/- FOXP3+ T cells (J, n = 17 STA and 15 REJ), 78.45% PD-1+ cells in FOXP3+CD8+CD45RClo/- T cells (K, n = 16 STA and 13 REJ), and 10.78% HLA-DR+ cells in CD8+CD45RClo/- FOXP3- T cells (L, n = 16 STA and 13 REJ) after transplantation. (D, F-L) Log-rank (Mantel Cox) test, **p<0.01, ***p<0.001. (M) ROC curves illustrating the specificity and sensitivity of the diagnosis of rejection based on the expression of CD28, PD-1, HLA-DR, CD103, CD154, GITR, and IFNγ, on FOXP3+CD45RClo/-CD4+ and CD8+ Tregs. (PDF) [file pone.0300032.s002.pdf]

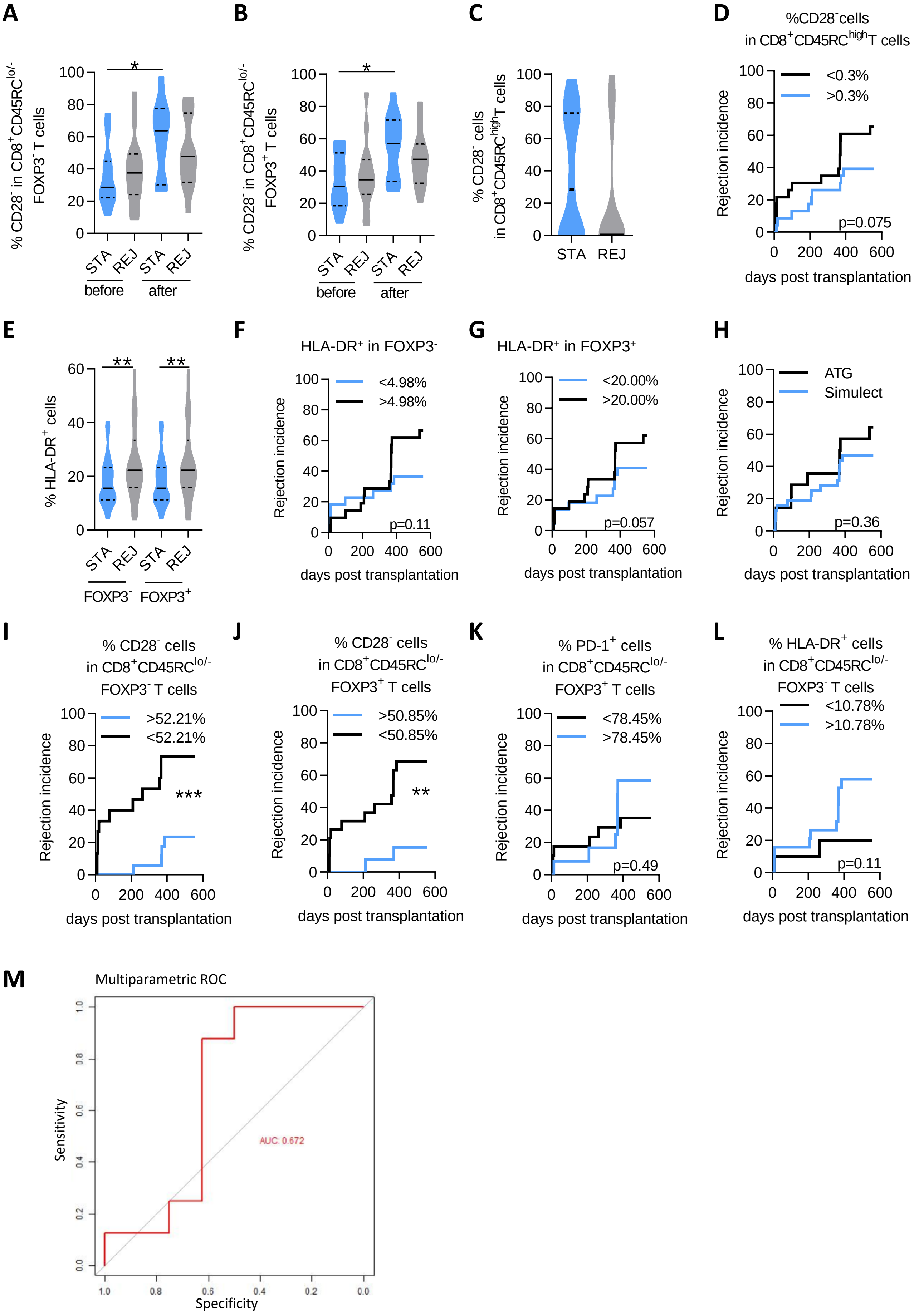
A
% CD28- in CD8+CD45RClo/- FOXP3- T cells
*
STA REJ STA REJ
before after
B
% CD28- in CD8+CD45RClo/- FOXP3+ T cells
*
STA REJ STA REJ
before after
C
% CD28- cells in CD8+CD45RChigh T cells
STA REJ
D
%CD28- cells in CD8+CD45RChigh T cells
<0.3%
>0.3%
p=0.075
Rejection incidence
days post transplantation
E
% HLA-DR+ cells
**
**
STA REJ STA REJ
FOXP3- FOXP3+
F
HLA-DR+ in FOXP3-
<4.98%
>4.98%
p=0.11
Rejection incidence
days post transplantation
G
HLA-DR+ in FOXP3+
<20.00%
>20.00%
p=0.057
Rejection incidence
days post transplantation
H
ATG
Simulect
p=0.36
Rejection incidence
days post transplantation
I
% CD28- cells in CD8+CD45RClo/- FOXP3- T cells
>52.21%
<52.21%
***
Rejection incidence
days post transplantation
J
% CD28- cells in CD8+CD45RClo/- FOXP3+ T cells
>50.85%
<50.85%
**
Rejection incidence
days post transplantation
K
% PD-1+ cells in CD8+CD45RClo/- FOXP3+ T cells
<78.45%
>78.45%
p=0.49
Rejection incidence
days post transplantation
L
% HLA-DR+ cells in CD8+CD45RClo/- FOXP3- T cells
<10.78%
>10.78%
p=0.11
Rejection incidence
days post transplantation
M
Multiparametric ROC
AUC: 0.672
Sensitivity
Specificity

**S2 Fig. Correlation of CD28, PD-1 and HLA-DR expression in CD8$^{+}$CD45RC$^{lo/-}$ T cell subsets and IS treatment with graft outcome.**

**(A-B)** Frequency of CD28$^{-}$ cells in CD8$^{+}$CD45RC$^{lo/-}$ FOXP3$^{-}$ **(A)** and FOXP3$^{+}$ **(B)** T cells in STA (n=11) and REJ (n=13) patients before and after transplantation. Wilcoxon matched-pairs signed rank test for time comparison and Mann Whitney test for groups comparison, *p<0.05. **(C)** Frequency of CD28$^{-}$ cells in CD8$^{+}$CD45RC$^{hi}$ T cells. STA, n=22; REJ, n=24. **(D)** Incidence of allograft rejection for patients having more (blue line) or less (black line) than 0.3% CD28$^{-}$ cells in CD8$^{+}$CD45RC$^{hi}$ T cells. **(E)** Frequency of HLA-DR$^{+}$ cells in FOXP3$^{-}$ and FOXP3$^{+}$ CD4$^{+}$CD45RC$^{lo/-}$ T cells. STA, n=20; REJ, n=23. Mann Whitney test, **p<0.01. **(F-G)** Incidence of allograft rejection for patients having more (black line) or less (blue line) than 4.98% HLA-DR$^{+}$ cells in CD4$^{+}$CD45RC$^{lo/-}$ FOXP3$^{-}$ T cells (**F**) or 20% HLA-DR$^{+}$ cells in CD4$^{+}$CD45RC$^{lo/-}$ FOXP3$^{+}$ T cells **(G)**. **(H)** Incidence of allograft rejection for patients treated with Simulect (n=32) or ATG (n=14) as induction. **(I-L)** Incidence of allograft rejection for patients treated with Simulect and having more (blue line) or less (black line) than 52.21% CD28$^{-}$ cells in CD8$^{+}$CD45RC$^{lo/-}$FOXP3$^{-}$ T cells (**I,** n=17 STA and 15 REJ), 50.85% CD28$^{-}$ cells in CD8$^{+}$CD45RC$^{lo/-}$ FOXP3$^{+}$ T cells (**J,** n=17 STA and 15 REJ), 78.45% PD-1$^{+}$ cells in FOXP3$^{+}$CD8$^{+}$CD45RC$^{lo/-}$ T cells (**K,** n**=**16 STA and 13 REJ), and 10.78% HLA-DR$^{+}$ cells in CD8$^{+}$CD45RC$^{lo/-}$ FOXP3$^{-}$ T cells (**L,** n**=**16 STA and 13 REJ) after transplantation. **(D, F-L)** Log-rank (Mantel Cox) test, **p<0.01, ***p<0.001. **(M)** ROC curves illustrating the specificity and sensitivity of the diagnosis of rejection based on the expression of CD28, PD-1, HLA-DR, CD103, CD154, GITR, and IFNγ, on FOXP3$^{+}$CD45RC$^{lo/-}$CD4$^{+}$ and CD8$^{+}$ Tregs.
