# Supplementary material for: Renal graft function in transplanted patients correlates with CD45RC T cell phenotypic signature
Source: PLoS One. 2024 Mar 21;19(3):e0300032. doi: 10.1371/journal.pone.0300032 (PMC10956768; doi:10.1371/journal.pone.0300032)
Supplement: S3 Fig — Incidence of graft rejection in patients that display more (black line) or less (blue line) than 40.29% or 47.63% PD-1+ cells in CD4+ (A) or CD8+ (B) T cells before transplantation. n = 47. Log Rank (Mantel Cox) test, ns. (PDF) [file pone.0300032.s003.pdf]

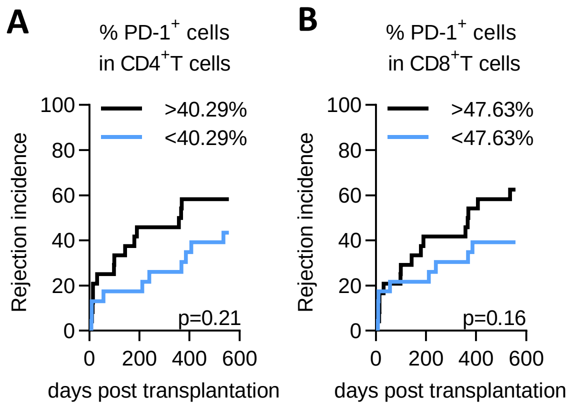

S3 Fig. PD-1 expression in CD4⁺ and CD8⁺ T cells before transplantation is not predictive of graft outcome.

Incidence of graft rejection in patients that display more (black line) or less (blue line) than 40.29% or 47.63% PD-1⁺ cells in CD4⁺ **(A)** or CD8⁺ **(B)** T cells before transplantation. n=47. Log Rank (Mantel Cox) test, ns.
