# Supplementary material for: Renal graft function in transplanted patients correlates with CD45RC T cell phenotypic signature
Source: PLoS One. 2024 Mar 21;19(3):e0300032. doi: 10.1371/journal.pone.0300032 (PMC10956768; doi:10.1371/journal.pone.0300032)
Supplement: S1 Table — (PDF) [file pone.0300032.s004.pdf]

S1 Table. mAbs used for immunophenotyping of patients by flow cytometry.

Marker	Provider	Clone
CD28	BD Biosciences	CD28.2
CD3	BD Biosciences	UCHT1
CD45RC	IQProduct	MT2
CD4	BD Biosciences	SK3
CD27	BD Biosciences	M-T271
CD45RA	BD Biosciences	HI100
Foxp3	BD Biosciences	259D/C7
IL-10	BD Biosciences	JES3-9D7
IL-34	R&D Biotechne	# 578416
IFNγ	BD Biosciences	B27
GITR	Miltenyi Biotec	DT5D3
CD154	BD Biosciences	TRAP1
TGF-β1	BD Biosciences	TW4-9E7
T-bet	BD Biosciences	O4-46
CD297 PD-1	BD Biosciences	EH12.1
CD103	BD Biosciences	Ber-ACT8
HLADR	BD Biosciences	L243
CD127	BD Biosciences	HIL-7R-M21
