# Supplementary material for: Renal graft function in transplanted patients correlates with CD45RC T cell phenotypic signature
Source: PLoS One. 2024 Mar 21;19(3):e0300032. doi: 10.1371/journal.pone.0300032 (PMC10956768; doi:10.1371/journal.pone.0300032)
Supplement: S1 Graphical abstract — (PDF) [file pone.0300032.s005.pdf]

Renal graft function in transplanted patients correlates with CD45RC T cell phenotypic signature

Patient cohort

- Retrospective study
- Stratification on clinical parameters
- 69 patients

	Before Tx	After Tx	
Stable graft function	n=23	M+12<br>n=22	n=44
≥1 Rejection episode	n=24	tREJ<br>n=24	n=45
	n=47	n=46	n=93 samples

2 time points of blood sampling

Methodology

CD4⁺ and CD8⁺ T cell subsets

↓

Segregation on CD45RC and Foxp3 expression

↓

Fluorescent-based high dimensional immunophenotyping

↓

- Correlations/clinic
- ROC curves

individual markers

↓

Multi parametric ROC

Signature

**CD45RC$^{lo/-}$ Treg**	Foxp3	CD127<br>GITR	CD103<br>CD154<br>CD45RA	CD28<br>TGF-β<br>↑PD1<br>↑HLA-DR
**Tconv**	IL-34	PD1	GITR<br>↓CD45RC<br>↓ CD28<br>↓CD127<br>↓T-bet	IL-34<br>↑PD1<br>↑HLA-DR<br>↑ IFNγ

Cell segregation based on the CD45RC marker and combination of multiple markers associated with inflammation or Tregs/tolerance allowed to identify the signature of stable graft function.
