# Supplementary material for: Renal graft function in transplanted patients correlates with CD45RC T cell phenotypic signature
Source: PLoS One. 2024 Mar 21;19(3):e0300032. doi: 10.1371/journal.pone.0300032 (PMC10956768; doi:10.1371/journal.pone.0300032)
Supplement: Attachment — Submitted filename: PONE-D-23-20905.docx [file pone.0300032.s006.docx]

The manuscript, Bézie et al., report a CD45RC T cell phenotypic signature associated with the outcome of kidney transplanted patients. This study implies the importance of biomarkers to monitor the transplant outcomes in study participant groups (STA vs. REJ). The results are very interesting, and the conclusions were appropriately supported. However, the major area to improve readability would be to emphasize the translation of these findings into clinical applications. The manuscript can be accepted for publication after addressing minor revisions.

- The role of PD-1 for Tregs should be elaborated with relevance to graft rejection and how it is correlated with the findings of this study.
- How does PD-1 expression on CD8^+^CD45RC^lo/-^ and CD8^+^CD45RC^hi^ T cells before and after transplantation correlate with a poorer outcome? Elaborate.
- The effect of anti-CD45RC therapy on kidney transplantation outcomes should be discussed.
- A graphical abstract representing the overall findings will add readability.
- The conclusions and future direction of this study should be discussed in detail.
