# Supplementary material for: Renal graft function in transplanted patients correlates with CD45RC T cell phenotypic signature
Source: PLoS One. 2024 Mar 21;19(3):e0300032. doi: 10.1371/journal.pone.0300032 (PMC10956768; doi:10.1371/journal.pone.0300032)
Supplement: Attachment — Submitted filename: Response to Reviewers PlosOne.docx [file pone.0300032.s007.docx]

The manuscript, Bézie et al., report a CD45RC T cell phenotypic signature associated with the outcome of kidney transplanted patients. This study implies the importance of biomarkers to monitor the transplant outcomes in study participant groups (STA vs. REJ). The results are very interesting, and the conclusions were appropriately supported. However, the major area to improve readability would be to emphasize the translation of these findings into clinical applications. The manuscript can be accepted for publication after addressing minor revisions.

**Answer**: **We thank the reviewers and editors for acknowledging the quality of our study.**

- The role of PD-1 for Tregs should be elaborated with relevance to graft rejection and how it is correlated with the findings of this study.
- How does PD-1 expression on CD8^+^CD45RC^lo/-^ and CD8^+^CD45RC^hi^ T cells before and after transplantation correlate with a poorer outcome? Elaborate.

**Answer**: **We added a few points to the discussion: “Interestingly, PD-1 was also reported being expressed by exhausted human CD8^+^HLA-DR^+^ Tregs (*1*) and dysfunctional CD4^+^ Tregs (*2*). The high frequency of HLA-DR^+^ cells within CD8^+^ and CD4^+^ Tregs we observed in the cohort after transplantation correlating with graft rejection supports the hypothesis of exhausted unfunctional Tregs in rejecting patients. Anti-PD-1 mAb treatment is evaluated in patients to stimulate activation of tumor reactive T cells, however it also stimulates PD1^+^ CD4^+^ and CD8^+^ Tregs resulting in higher Treg suppressive activity and unwanted progression of cancer (*3*). While anti-PD-1 mAbs might be beneficial for transplanted patients at one year post transplant to stimulate Tregs, the high frequency of activated PD-1^+^ (and HLA-DR^+^) cells in CD8^+^CD45RC^hi^ T cells before the transplantation correlating with poor graft outcome, argues for a later application.”**

- The effect of anti-CD45RC therapy on kidney transplantation outcomes should be discussed.

**Answer**: **We have now added a discussion: “Targeting CD45RC with a depleting mAb showed high efficacy to establish tolerance in models of heart transplantation, GvHD, APECED and Duchenne’s dystrophy ^4-7^. The short-term effect was mediated by pathogenic cell depletion, while the long-term therapeutic effect in these models was mediated by Tregs of increased suppressive activity able to induce infectious tolerance. Indeed, since CD45RC is the only CD45 isoform not expressed by Tregs, it represents a promising therapeutic to target non Tregs in transplant patients.”**

- A graphical abstract representing the overall findings will add readability.

**Answer**: **We thank the reviewer for his advice, a graphical abstract was added.**

- The conclusions and future direction of this study should be discussed in detail.

**Answer: We now discuss:**

**“Recently developed multiproteomic analysis of pan T lymphocytes in tissue biopsies makes it possible to deeply characterize the T cell infiltrate of the graft. Our efforts will now focus on finding correlations between graft-infiltrating and blood T cells profile in order to find more precise and relevant blood diagnostic markers of the response to the allograft. In addition, a better understanding of the kinetics of response to allograft by CD8^+^ T cell subpopulations will allow real-time diagnosis.”**

References:

1.	A. Machicote, S. Belén, P. Baz, L. A. Billordo, L. Fainboim, Human CD8+HLA-DR+ Regulatory T Cells, Similarly to Classical CD4+Foxp3+ Cells, Suppress Immune Responses via PD-1/PD-L1 Axis. *Front. Immunol.* **9**, 2788 (2018).

2.	D. E. Lowther, B. A. Goods, L. E. Lucca, B. A. Lerner, K. Raddassi, D. Van Dijk, A. L. Hernandez, X. Duan, M. Gunel, V. Coric, S. Krishnaswamy, J. C. Love, D. A. Hafler, PD-1 marks dysfunctional regulatory T cells in malignant gliomas. *JCI Insight* **1** (2016).

3.	T. Kamada, Y. Togashi, C. Tay, D. Ha, A. Sasaki, Y. Nakamura, E. Sato, S. Fukuoka, Y. Tada, A. Tanaka, H. Morikawa, A. Kawazoe, T. Kinoshita, K. Shitara, S. Sakaguchi, H. Nishikawa, PD-1 ^+^ regulatory T cells amplified by PD-1 blockade promote hyperprogression of cancer. *Proc. Natl. Acad. Sci.* **116**, 9999–10008 (2019).

4.	Boucault L, Lopez Robles MD, Thiolat A, et al. Transient antibody targeting of CD45RC inhibits the development of graft-versus-host disease. Blood Adv. 2020;4(11):2501-2515. doi:10.1182/bloodadvances.2020001688

5.	Ouisse LH, Remy S, Lafoux A, et al. Immunophenotype of a Rat Model of Duchenne’s Disease and Demonstration of Improved Muscle Strength After Anti-CD45RC Antibody Treatment. Front Immunol. 2019;10:2131. doi:10.3389/fimmu.2019.02131

6.	Besnard M, Sérazin C, Ossart J, et al. Anti-CD45RC antibody immunotherapy prevents and treats experimental Autoimmune PolyEndocrinopathy Candidiasis Ectodermal Dystrophy syndrome. J Clin Invest. Published online February 15, 2022. doi:10.1172/JCI156507

7.	Picarda E, Bézie S, Boucault L, et al. Transient antibody targeting of CD45RC induces transplant tolerance and potent antigen-specific regulatory T cells. JCI Insight. 2017;2(3):e90088. doi:10.1172/jci.insight.90088
