# Supplementary material for: Renal graft function in transplanted patients correlates with CD45RC T cell phenotypic signature
Source: PLoS One. 2024 Mar 21;19(3):e0300032. doi: 10.1371/journal.pone.0300032 (PMC10956768; doi:10.1371/journal.pone.0300032)
Supplement: Attachment — Submitted filename: PONE-D-23-20905R1.docx [file pone.0300032.s008.docx]

Dear Editor-in-chief,

The authors have revised the manuscript as per the suggestions. The manuscript can be accepted for publication in PLOS ONE.
