# Supplementary material for: Renal graft function in transplanted patients correlates with CD45RC T cell phenotypic signature
Source: PLoS One. 2024 Mar 21;19(3):e0300032. doi: 10.1371/journal.pone.0300032 (PMC10956768; doi:10.1371/journal.pone.0300032)
Supplement: Attachment — Submitted filename: Response to reviewer PONE-D-23-20905R1 2nd review.docx [file pone.0300032.s009.docx]

Dear Editor-in-chief,

The authors have revised the manuscript as per the suggestions. The manuscript can be accepted for publication in PLOS ONE.

6. Review Comments to the Author

Please use the space provided to explain your answers to the questions above. You may also include additional comments for the author, including concerns about dual publication, research ethics, or publication ethics. (Please upload your review as an attachment if it exceeds 20,000 characters)

Reviewer #1: Thanks to all authors for the intersting work. The study is interesting with pre transplantation data and a good follow up period of 4.5 years.
I have the following points:

1. In the introduction, authors stated how the current biomarkers used to predict outcome are costly and slow to implement. It would be better to reflect on how results of the current study address these issues in the discussion.

**Answer: We added in the discussion: “****These results show that there is no dichotomous marker making it possible to predict the outcome of the transplant but a combination of markers defining the state of activation of cell subsets providing a probability on the status of the graft and of the outcome of the transplant. (…) Some markers are already clinically investigated and we propose adding at least the CD45RC marker, or even FOXP3, to add value to each of the validated markers without disrupting all the protocols.”**

2. In the methodology, authors stated that 50% of the cohort had AR! This is a bit high! Is there any explantaion? or it is the selection process? Better to clarify.

**Answer: Among the 300 patients included in the DIVAT biocollection, 34.7% had an AR in the first 18 months following the transplant. For this study, we selected patients such that 50% had AR and 50% did not. We clarified in the material and methods section: “This retrospective study is based on first transplanted patients selected from the Nantes DIVAT biocollection such that 50% had acute~~, with a~~ rejection occurring within the first 18 months and 50% did not ~~for 50% of the cohort~~.”**

3. What about the pretransplant sensitization history and data of the cohort?

**Answer: We indicated in Table 1 that 8.7% of selected patients were secondly transplanted.**

4. Was there any relation between the trajectory of GFR (40-60 OR >60), and proteinuria (0 or <0.5 gm) with the study results?

**Answer: These criteria were used to identify STA and REJ patients, so a correlation of immune markers with graft rejection assumes a correlation with these clinical markers. We defined in the material and methods section that “A stable function of the graft is defined as stable creatinine below 150 µmol/L (ideally < 100 µmol/L), zero proteinuria or less than 0.5 g/24h or g/g, an immunosuppressive treatment other than Sirolimus (Rapamune) or Everolimus (Certican), a clearance greater than 40 ml/min in MDRD and no DSA for more than one year.”**

5. Any correlation with CNI trough levels as IS may have a differential effect in STA vs. REJ patients?

**Answer: The reviewer is right, all patients were treated with CNI, CsA and TAC as shown in Table 1 with doses adapted to their immune status. We added in the material and method section that “****CNI doses were adapted according to the clinical criteria for graft rejection in order to minimize side effects. Therefore, patients defined as STA received lower doses of CNI than REJ patients.”**

6. More elaboration about the role of PD-1 needed.

**Answer: We are already discussing significantly on the role of PD-1 with new sections added following the 1st review, we believe that it would unbalance the discussion to add more elaboration on this marker only vs the other markers: “PD-1 and HLA-DR expression on CD4^+^ and CD8^+^ total T cells did not correlate with graft outcome in this cohort either, but their high expression on CD8^+^CD45RC^lo/-^FOXP3^+^ and FOXP3^-^ Tregs respectively after transplantation and of PD-1 on CD8^+^CD45RC^hi^ T cells before transplantation positively correlated with a poorer outcome. The role of PD-1 for Tregs is controversial. PD-1 has been reported being involved in CD4^+^Tregs generation and suppressive properties ^28^, to identify regulatory cells versus memory cells among CD8^+^ Tregs ^29^, and notably PD-1^+^CD122^+^CD8^+^ Tregs have been shown to prolong murine allograft survival ^30^. By contrast, other studies reported that PD-1 deficient CD4^+^ Tregs were more functional as inhibiting efficiently experimental autoimmune encephalomyelitis or diabetes in mice ^31^.** **(…) Interestingly, PD-1 was also reported being expressed by exhausted human CD8^+^HLA-DR^+^ Tregs ^32^ and dysfunctional CD4^+^ Tregs ^34^. (..) Anti-PD-1 mAb treatment is evaluated in patients to stimulate activation of tumor reactive T cells, however it also stimulates PD1^+^ CD4^+^ and CD8^+^ Tregs resulting in higher Treg suppressive activity and unwanted progression of cancer ^35^. While anti-PD-1 mAbs might be beneficial for transplanted patients at one year post transplant to stimulate Tregs, the high frequency of activated PD-1^+^ (and HLA-DR^+^) cells in CD8^+^CD45RC^hi^ T cells before the transplantation correlating with poor graft outcome, argues for a later application.”**

7. It would be better if there is data about any correlation with elements of humoral response or correlation with biopsy proven AMR or any MVI!?

**Answer: The cohort of patients was constituted in such a way as to identify markers reflecting the stable or inflammatory state of the graft such that all patients presenting humoral responses were included in the REJ group and none in the STA group. Therefore, markers correlated with stable graft function may not correlate with humoral response. We defined in the material and methods section that “Rejection was defined by humoral, cellular or borderline rejection according to Banff classification in effect at the time of diagnosis proven by biopsy. None of these signs were observed in systematic biopsy on month+12 in stable patients”. In addition, the cohort constituted does not include enough patients who developed a humoral response to study the correlation of markers with this subgroup.**

6. Better to add the study limitation.

**Answer: We indicated in the discussion section that “This [result] may be due to the smaller cohort of patients compared to what was used previously by other groups”, and we added “We focused our efforts on the investigation of the Teff/Treg balance and profile through broad immunophenotyping however applied to a limited number of patient samples, and were unable to increase the resolution of the prediction to the type of cellular or humoral rejection within the REJ group.”**

7. Yes, a graphical abstract will add a lot.

**Answer: A graphical abstract was added and submitted to PlosOne.**
